# The Perceived Benefits of Height: Strength, Dominance, Social Concern, and Knowledge among Bolivian Native Amazonians

**DOI:** 10.1371/journal.pone.0035391

**Published:** 2012-05-04

**Authors:** Eduardo A. Undurraga, Leslie Zebrowitz, Dan T. A. Eisenberg, Victoria Reyes-García, TAPS Bolivia Study Team, Ricardo A. Godoy

**Affiliations:** 1 Heller School for Social Policy, Brandeis University, Waltham, Massachusetts, United States of America; 2 Department of Psychology, Brandeis University, Waltham, Massachusetts, United States of America; 3 Department of Anthropology, Northwestern University, Evanston, Illinois, United States of America; 4 Department of Anthropology, University of Washington, Seattle, Washington, United States of America; 5 ICREA and Institut de Ciència i Tecnologia Ambientals, Universitat Autònoma de Barcelona, Bellaterra, Barcelona, Spain; 6 Tsimane’ Amazonian Panel Study (TAPS), San Borja, Beni, Bolivia; Indiana University, United States of America

## Abstract

Research in industrial countries suggests that, with no other knowledge about a person, positive traits are attributed to taller people and correspondingly, that taller people have slightly better socioeconomic status (SES). However, research in some non-industrialized contexts has shown no correlation or even negative correlations between height and socioeconomic outcomes. It remains unclear whether positive traits remain attributed to taller people in such contexts. To address this question, here we report the results of a study in a foraging-farming society of native Amazonians in Bolivia (Tsimane’)–a group in which we have previously shown little association between height and socioeconomic outcomes. We showed 24 photographs of pairs of Tsimane’ women, men, boys, and girls to 40 women and 40 men >16 years of age. We presented four behavioral scenarios to each participant and asked them to point to the person in the photograph with greater strength, dominance, social concern, or knowledge. The pairs in the photographs were of the same sex and age, but one person was shorter. Tsimane’ women and men attributed greater strength, dominance, and knowledge to taller girls and boys, but they did not attribute most positive traits to taller adults, except for strength, and more social concern only when women assessed other women in the photographs. These results raise a puzzle: why would Tsimane’ attribute positive traits to tall children, but not tall adults? We propose three potential explanations: adults’ expectations about the more market integrated society in which their children will grow up, height as a signal of good child health, and children’s greater variation in the traits assessed corresponding to maturational stages.

## Introduction

Taller people in industrial countries are attributed positive socioeconomic and character traits, such as intelligence, employability, and leadership [1]–[4]. Additionally, height is positively associated with various socioeconomic indicators of well-being such as wealth, income, education, happiness, and success [5]–[16]. These results may be partly explained because height influences our perceptions of others and how others perceive and react to us [17]–[19], and because tallness might signal unobserved traits related to productivity (e.g., strength, self-esteem) [20], [21].

Research in industrial countries has also addressed how gender mediates perceptions of height. Results suggest that positive attributions to tall men (e.g., intelligence, affluence, dominance) also apply to tall women [1], [2]. Taller women are also perceived as more masculine and less expressive or caring, particularly by men [1], [22]. Research in the USA suggests that parents and teachers view taller children (particularly boys) as more competent than shorter children of the same age and sex [23]–[25]. Although the height premium is probably not linear–being too tall might be associated with worse outcome–for most of the height distribution greater height seems to be associated with better outcomes [26].

But the universality of these findings has been contested by research in developing countries [9], [27], [28]. Consistent with findings in industrial countries, on average, taller men and women earned higher wages in urban Brazil [21], in rural Philippines [29], and in Indian coal mines [30], and taller Indian children did better in cognitive tests [31]. However, height was negatively correlated with foraging productivity among the !Kung San in Africa [32], and among the Tsimane’ in the Bolivian Amazon adult height bore no association with many socioeconomic indicators of well-being, including schooling, income, or wealth [33]. Sorokowski et al. found that semi-nomadic pastoralist women in Namibia reported no preference for taller men [34]. A recent review of the cross-cultural evidence between height and reproductive success found much variation, concluding that “while short height is rarely advantageous, particularly for men, tall height is not universally beneficial, particularly for women” [27].

This mixed evidence raises a question: Do people across cultures attribute positive traits to the tall, or is the perception restricted to places where height correlates positively with socioeconomic outcomes? Studies in developing countries suggest that height bears a (weak) correlation with socioeconomic outcomes, but we do not know how well perceptions of height map onto socioeconomic realities.

If people across cultures attribute positive traits to tallness, even when tallness is not associated with increased socioeconomic status or reproductive success, it would suggest a simple conserved preference for tallness–perhaps evidence that tallness was a durable indicator of reproductive success in our adaptively relevant evolutionary past [26], [35], [36]. On the other hand, if tallness preference only persists in contexts where height is correlated with higher reproductive or socioeconomic benefits, then this would be evidence for greater flexibility in human cognition. People and their emergent cultures strategically adjust what they value based on what garners most reproductive success in a particular political, social, and ecological context. This has been interpreted as adaptive cultural evolution, which is allowed by human phenotypic plasticity [37], [38].

Here we report the results of a study in a non-Western culture to answer this question. The study responds to growing interest in establishing the external validity of studies on height perception in industrial countries [20], [39]. Our study was conducted among the Tsimane’, a foraging-farming society in the Bolivian Amazon. The Tsimane’ number ∼8,000 people and live in ∼120 villages along river banks. Their mostly autarkic subsistence centers on hunting, fishing, and slash-and-burn agriculture. Tsimane’ live in a relatively egalitarian society and are highly endogamous, probably connected to their practice of preferential cross-cousin marriage [40]–[42]. Because the Tsimane’ have a very different lifestyle, values, and socioeconomic organization than those found in industrial nations, they provide an apt setting to further test whether the attribution of positive traits to tallness is universal.

This research area and our results also have implications for public health. Child growth stunting is widespread in rural areas of developing countries [43], including the Tsimane’ [44], [45]. If people do not attribute positive traits to tall children, one could argue that a contributor to child growth stunting would be cultural–parents may not give enough importance to growth faltering because they do not associate child height with desirable outcomes [46]. But if people assign positive attributes to tallness, the prevalence of growth faltering would suggest that the impediment to normal growth resides in other areas, such as poor nutrition and access to health care services, or low income.

### Hypotheses

Drawing on findings from industrial countries, we test three hypotheses:

Hypothesis-1: *Tsimane’ judges will perceive taller adult women and men as stronger, more dominant, and more knowledgeable than shorter adults.*

Hypothesis-2: *Shorter women will be perceived as more caring than taller women, particularly by male raters*. We also explore whether shorter men are perceived as more caring than taller men.

Hypothesis-3: *Adults will evaluate taller children as stronger, more competent, and more knowledgeable than shorter children of the same sex and age.*

These hypotheses predict that Tsimane’ will attribute the same traits to height as do people in industrial countries. However, prior research among the Tsimane’ suggests that adult height bears weak associations with socioeconomic outcomes [33]. This inconsistency raises a question we aim to address: Do perceptions of height mirror the actual benefits of height in a society (in which case Tsimane’ should not value adult height) or do Tsimane’ preferences for tallness resemble the preferences found in industrial nations?

## Materials and Methods

### Ethics Statement

**Figure 1:**
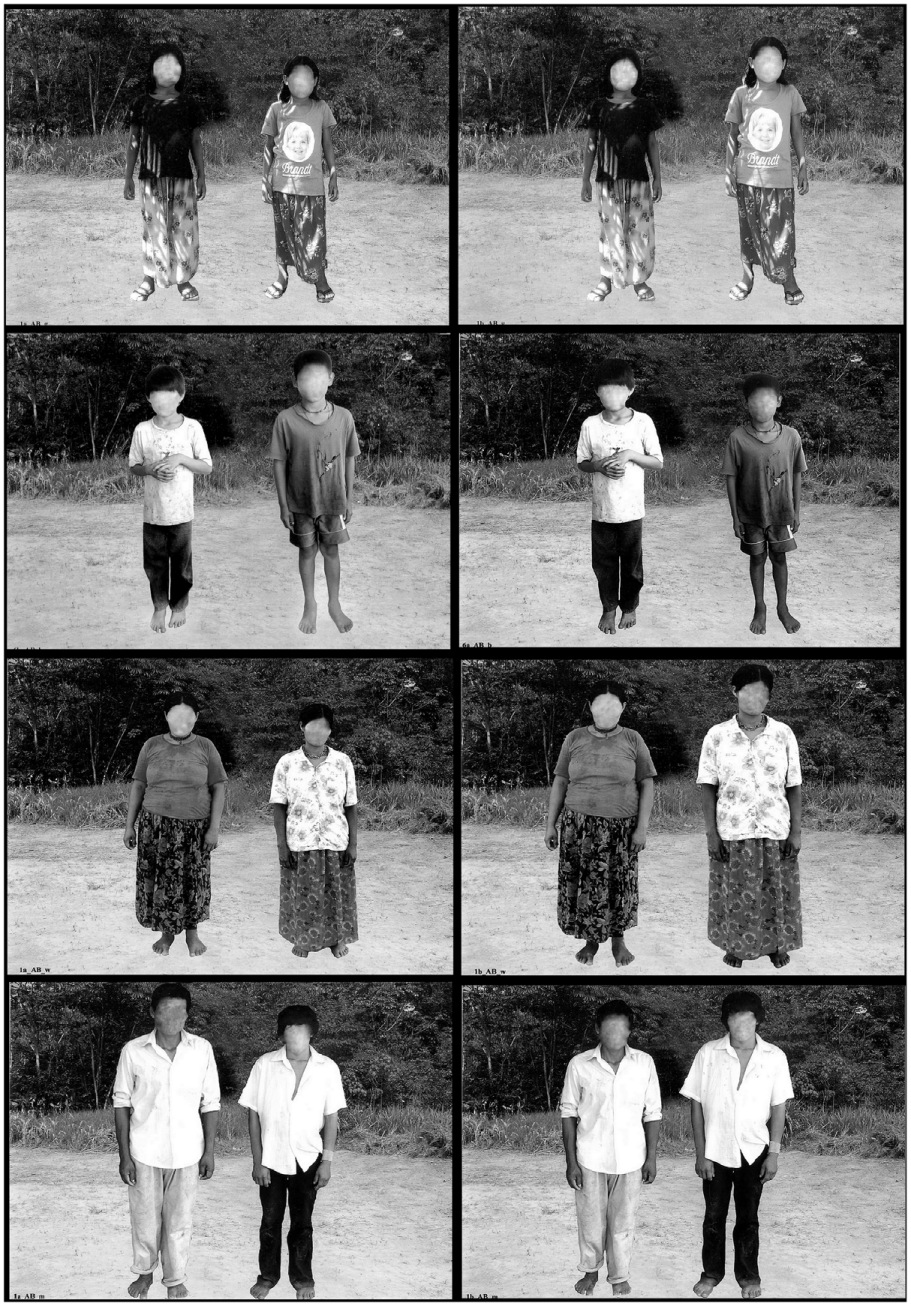
Example of photographs used: pairs of girls, boys, women, and men and comparison with the photograph alternating the tallest participant.

Due to the low levels of literacy of the Tsimane’, we obtained oral consent from the participants before enrollment to have their photograph taken and shown to others as part of this study. No Tsimane’ declined to participate in the study. The study received IRB approval from Brandeis University and from the Great Tsimane’ Council, the governing body of the Tsimane’. The publication of the photographs (Figure 1) in any scientific journal, with intentionally blurred faces to protect anonymity, also received approval from the Great Tsimane’ Council and from the IRB office of Brandeis University.

### Overview

During June-July, 2010, we sequentially showed 24 color photographs (15×10 cm) in an album to 40 women and to 40 men >16 years of age. Each photograph was on a separate page and showed two Tsimane’ of the same sex and ostensibly of the same age and body type standing side by side against the same background. The people in the photograph differed in height and were unknown to participants. Surveyors described a short behavioral scenario related to a desirable trait, and then asked the participant to point to the person in the photograph who could most easily do the task described. After the participant answered the question for all 24 photographs, the surveyor described a different behavioral scenario related to another trait, and repeated the procedure showing the 24 photographs in the same order.

### Stimuli: The Photographs

During 2009, we photographed Tsimane’ individually in a village about a day away from the area where the experiment would later take place. In this way, we reduced the likelihood that participants would know the people in the photographs. The people in the photographs had a neutral facial expression, and wore normal attire, without hats, sweaters, or jackets.

We prepared the photographs using Adobe Photoshop. First, we combined two separate photographs of two people of the same sex and roughly the same age into one photograph. Second, we edited the photographs so that all the photographs had the same background. People in the photographs appeared standing in a patch of clear land with forest vegetation in the background. We selected this familiar background to allow participants to judge the height of people in the photograph relative to an objective marker (e.g., vegetation), and focus on the two people. Third, in one set of photographs (n = 24) depicting pairs of people (A and B) we made person *A* taller than person *B* (A_B_). We then produced a second identical set of photographs (n = 24) depicting the same pairs, but in which person *A* was shorter than person *B* (_A_B).

We photographed women, men, girls, and boys. Because many Tsimane’ do not know their exact age [47], we used self-reported age and our own judgment in selecting Tsimane’ who were obvious adults or children. We excluded people who looked like teenagers or young adults and the elderly, to produce four sets of photographs depicting distinct, non-overlapping demographic groups. Figure 1 shows an example of the photographs (A_B_, _A_B) we used. Faces in Figure 1 have been intentionally blurred to protect the anonymity of the people photographed (but were not blurred during the actual study).

### The Test: Assessing the 24 Photographs

Each demographic group (i.e., women, men, girls, and boys) had six photographs depicting different pairs of people. All six photographs were placed together, so participants had to answer a question about all six pairs of people in a specific demographic group (e.g., women) before answering the same question about another group (e.g., men, girls, or boys). No rater saw the same people depicted with different heights. There were eight test orders created by crossing two photograph sets that varied which of the two people was taller (A_B_ or _A_B) with four orders for the demographic groups (e.g., women, men, girls, boys). Within a demographic group, the order in which raters saw the six photographs was random; three photographs had the taller person standing on the left (A_B_), and three photographs had the taller person standing on the right (_A_B).

For the tests, we first narrated a behavioral scenario about a trait and then asked a question about that trait [48]. Overall, the surveyor asked about four specific traits: (1) physical strength, (2) dominance, (3) social concern, and (4) ethno-medicinal plant knowledge (hereafter knowledge; for the importance and ubiquity of ethno-medicinal knowledge among Tsimane’ see Reyes-García et al. [49]). We chose the four traits based on previous studies and thus facilitate cross-cultural comparisons. We next use strength (1) to illustrate how we asked the questions.

Pointing to the first photograph in a test, the surveyor said “Look at these two people. There is a heavy bag with rice in the patio [open area outside the house] and it is going to rain. Who of the two is stronger and could bring the bag inside the house faster?” If the question was about children in the photographs, the surveyor prefaced the question with: “Look at these two children. They are of the same age”. Participants then pointed to one person in the photograph. If the participant could not decide, surveyors pressed participants to make a choice between the two. We analyze results with and without induced answers. The surveyor used the following phrasing to elicit answers about the other traits: (2) Dominance: “Look at these two people. They want to spend leisure time together, but one of them wants to take a walk, while the other wants to go fishing. Who of the two is going to decide what to do?” (3) Concern: “Look at these two people. They find a juvenile/infant monkey in the old-growth forest. Who of the two will take better care of the monkey?” (4) Knowledge: “Look at these two people. They are trying to find a plant in the old-growth forest to cure diarrhea. Who of the two will know better which plant to use?”.

At the end of the test, the surveyor asked participants if they knew any of the people in the photographs, and again showed them the 24 photographs. Only two participants knew people in the photographs; one person knew 10 people and the other knew three people. For these two subjects, these photographs were dropped from the analysis.

### Administration of the Experiment

Most of the raters (n = 73) lived in the village of Santa Maria, but seven lived in the nearby village of Maraca. We included these participants because Santa Maria did not have 80 eligible persons. The mean and median age of the 40 women were 35 and 29 years (standard deviation [SD] = 18; range: 16–80) and of the 40 men were 35 and 31 years (SD = 17; range: 16–89). We asked all adults in Santa Maria to rate the photographs, and randomly selected seven adults from the village of Maraca. All but one of the participants was part of a nine-year longitudinal study with the Tsimane’ [50]. For participating in the study, women received wool, soap, a metal knife, sugar, and a topical medical ointment, and men received flashlight batteries, bullets, fishing line, fishing hooks, and a cigarette lighter.

Santa Maria and Maraca lie along the Maniqui River (department of Beni), about six hours by canoe from the nearest market town (San Borja). It takes ∼4.5 hours to walk from Santa Maria to Maraca during the dry season. Santa Maria has 33 households and 158 inhabitants and Maraca has 12 households and 72 inhabitants. Santa Maria and Maraca have per capita daily monetary income of ∼$US0.40 and $US0.20 respectively. Tsimane’ practice cross-cousin marriage and frequently migrate between villages [51], and subsistence in all villages centers on slash and burn agriculture and foraging [51]–[53].

RG trained two Tsimane’ who had worked in the longitudinal study since its inception to administer the test. These surveyors knew the participants but were not told the purpose of the test, so it is unlikely that they influenced responses. Surveyors administered the test at the participants’ home. Tests were done outdoors to enhance the visibility of the photographs. Only during rainy days did we collect data inside the house. The experiment lasted ∼30 minutes (SD = 7 minutes; range:16–65).

### Analysis

We use the participant’s response to the photograph as the unit of analysis and outcome variable, and use a Linear Probability Model (LPM) to model responses as a function of four vectors of variables:
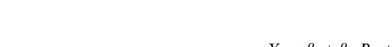



*Y* stands for the dichotomous response of participant *i* to photograph *p* (1 =  rater chooses taller person; 0 =  otherwise). *Participant* refers to the self-reported age (years) and sex of the person who rated the photograph. *Traits* include four dummy variables (*strength*, *dominance*, *social concern, knowledge*), with one excluded category in each regression, and refers to the type of question posed to the participant. *Demography* includes dummy variables capturing the four demographic groups (*men*, *women*, *boys,* and *girls*) of the people shown in the photographs, with the excluded category dependent on the hypothesis. *Controls* include variables about the photographs and the context of the test, such as a dummy for the position of the taller person (*Left;* 1 =  taller person on left; 0 =  otherwise), duration of the test (minutes), and a dummy variable for the participant’s village of residence. Because each participant had a maximum of 96 responses, we do the analysis clustering by participant. As an alternative specification to estimate the probabilities of choosing the taller person in the photograph, we could have used Logit or Probit regressions, but use LPM for ease of interpretation. We used Stata11 for the analysis.

## Results

**Figure 2:**
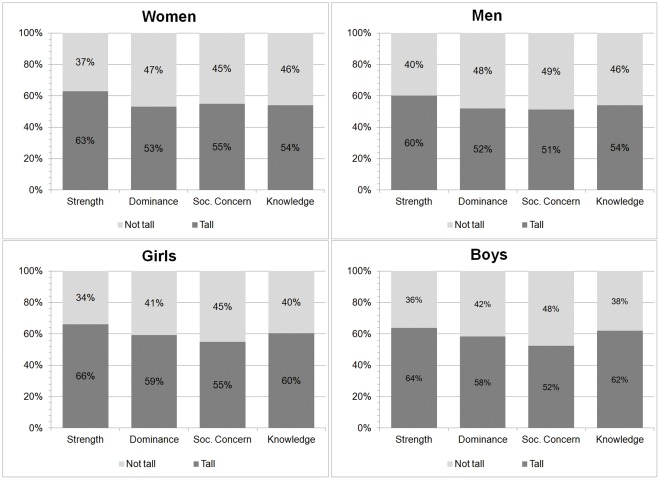
Distribution of responses for each trait by demographic group of person in photograph.

Figure 2 shows the distribution of responses by demographic group of the photographed individual and trait being rated. The figure suggests that Tsimane’ tend to attribute positive traits to the tall. We did two-sided binomial tests to examine whether the proportion of participants who chose the taller person in the photograph for each of the traits differed significantly from 50%. For strength, the proportion of participants who chose the taller person was significantly different from 50% for all demographic groups (p = 0.001). For dominance, the proportion differed significantly from 50% for children (girls and boys, p = 0.001), but not for adults (women, p = 0.20; men, p = 0.41). The percentage of participants who associated taller adult women and girls with greater social concern was less than 50% (adult women and girls, p = 0.03 in both), but the percentage of participants who associated taller adult men and boys with greater social concern was not significantly different from 50% (adult men, p = 0.61; boys, p = 0.34). Last, the share of participants who chose the taller person in the photograph when asked about knowledge differed significantly from 50% for children (girls and boys, p<0.001 in both), but only marginally significant for adults (women, p = 0.08; men, p = 0.09).

The aggregate analysis for all the categories combined (not shown in Figure 2) suggests that overall, ∼55% of participants pointed to the taller person in the photograph when assessing adults and ∼60% pointed to the taller person when assessing children. The association was statistically significant (Pearson χ^2^(1) = 14.8; p = 0.001). Participants pointed to the taller person when assessing adult women on 56% of their responses to the four questions, compared with ∼54% of their responses when assessing adult men (Pearson χ^2^(1) = 1.4; p = 0.23). For children, participants pointed to the taller child in ∼60% of their responses about girls, and in ∼59% of their responses about boys (Pearson χ^2^(1) = 0.51; p = 0.48). Of the four traits, participants were most likely to point to the tall person when the question was about strength (pooled 63%; adults 62%, children 65%, Pearson χ^2^(1) = 2.5; p = 0.12), followed by knowledge (pooled 58%; adults 54%, children 61%, Pearson χ^2^(1) = 9.9; p<0.01), dominance (pooled 56%; adults 53%, children 59%, Pearson χ^2^(1) = 7.7; p<0.01), and social concern (pooled 54%; adults 53%, children 54%, Pearson χ^2^(1) = 0.06; p = 0.81).

These findings are informative, but do not consider the role of other variables. Table 1 contains multivariate regression results, organized around the three hypotheses of this work. Because the regressions leave out a reference category the results may be difficult to interpret, so, for ease of interpretation, we also estimated the expected probabilities and 95% CI of the participant choosing the taller individual in the photographs for each trait (Table 2) [54].

**Table 1:** Estimates of the attribution of positive traits to taller persons using Linear Probability Models (LPM).

	Dependent variable: 1 = participant chose the taller person; 0 = otherwise					
Explanatory variables	*H1a*	*H1b*	*H2a*	*H2b*	*H3a*	*H3b*
[I]. STIMULI (photographs)						
[A] Traits:						
[i] Direct effects:						
*1-Strength*	Ref.	Ref.	0.09**	0.09**	0.11**	0.11**
			(0.03)	(0.03)	(0.03)	(0.03)
*2-Dominance*	–0.09**	–0.10*	–0.01	–0.01	0.05*	0.04
	(0.03)	(0.04)	(0.03)	(0.03)	(0.02)	(0.03)
*3-Social concern*	–0.08**	–0.08**	0.01	–0.03	Ref.	Ref.
	(0.02)	(0.03)	(0.03)	(0.04)		
*4-Knowledge*	–0.07**	–0.09**	Ref.	Ref.	0.08**	0.05
	(0.03)	(0.03)			(0.03)	(0.04)
[ii] Interaction effects (traits and sex of theperson in the photograph):						
1-Strength*men						0.00
						(0.04)
2-Dominance*men		0.02				0.02
		(0.04)				(0.03)
3-Social concern*men		–0.01		0.08		Ref.
		(0.04)		(0.06)		
4-Knowledge*men		0.03				0.04
		(0.04)				(0.03)
[B] Demographic group (photographs)						
*1-Women*	Ref.	Ref.				
*2-Men*	–0.02	–0.03				
	(0.02)	(0.03)				
*3-Boys*					–0.01	–0.03
					(0.02)	(0.03)
*4-Girls*					Ref.	Ref.
[II]. PARTICIPANTS						
[A] Direct effects:						
*1-Female*	0.07**	0.07**	0.09**	0.07*	0.03	-0.03
	(0.02)	(0.02)	(0.03)	(0.03)	(0.03)	(0.03)
[B] Interaction effects of participant’s sex (*female*)*trait:						
1-Female*Social concern				0.08		
				(0.055)		
[III]. ADDITIONAL INFORMATION						
[A] Constant	0.54**	0.54*	0.37**	0.38**	0.46**	0.47**
	(0.05)	(0.05)	(0.07)	(0.07)	(0.78)	(0.08)
[B] Observations	3,799	3,799	1,900	1,900	3,828	3,828
[D] Photographs	Women and men		Women		Girls and boys	

Notes: * and ** significant at 5% and1% level respectively. All regressions include a dummy variable for the position of the taller person (left or right side), duration of test, and village of residence. Regressions were estimated using robust standard errors (in parentheses) and clustering by subject. Ref. = reference category.

**Table 2:** Predicted probabilities [95% confidence interval] that participant will rate the taller person in the photograph more positively on each of the four rated traits than the shorter person.

	H1a	H1a	H2a	H3a	H3a
Sex of person in photograph:	Women	Men	Women	Girls	Boys
TRAITS:					
Strength	62.5.	60.6	62.9	65.6	64.4
[95% CI]	[57.9–67.0]	[55.9–65.2]	[57.8–68.1]	[60.1–70.0]	[59.6–69.2]
Dominance	53.5	51.6	53.1	59.4	58.3
[95% CI]	[49.3–57.7]	[48.0–55.1]	[48.3–57.8]	[55.3–63.5]	[54.1–62.4]
Social Concern	54.1	52.1	54.9	54.2	53.1
[95% CI]	[50.7–57.4]	[48.5–55.8]	[50.2–59.7]	[50.2–58.2]	[49.2–57.0]
Knowledge	55.0	53.1	54.1	61.8	60.6
[95% CI]	[50.4–59.5]	[49.5–56.7]	[48.9–59.3]	[57.3–66.2]	[55.9–65.4]

Notes: Probabilities were estimated for an average rater of ∼35 years of age and a survey duration of 30 minutes. A 50% predicted probability would be expected by chance.

Hypothesis-1: *Tsimane’ judges will perceive taller adult women and men as stronger, more dominant, and more knowledgeable than shorter adults*. Column H1a, section IAi1 (Table 1), suggests that compared to answers about strength, participants were 8 percentage-points less likely to select the taller adult when asked about social concern (p = 0.001), 9 percentage-points less likely to select the taller adult when asked about dominance (p = 0.002), and 7.5 percentage points less likely to select the taller adult when asked about knowledge (p = 0.005). We re-ran the regressions of Column H1a but leaving out of the regression, as the reference category, first the question about dominance, then knowledge, and last social concern (regressions not shown). These regressions suggest that participants were equally likely to select the taller or the shorter adult when asked about dominance, knowledge, or social concern. We only found a significant difference when we compared the probability of selecting the taller adults in the photograph as stronger with the probability of choosing the taller adults for the other traits. The ∼7–9 percentage-points greater propensity to select the taller adult as stronger, as compared to other traits, applied equally to photographs depicting women and to photographs depicting men [column H1a, section IAii].

To better illustrate the results, we also estimated the probabilities of choosing the taller person in the photographs for each trait, by the sex of the person shown in the photographs (Table 2). We computed the probabilities for an average rater of ∼35 years of age who took ∼30 minutes to complete the test. These results suggest that the predicted probability that the rater attributed more strength to taller individuals in photographs of women was 62.5% (95% CI: 57.9–67), and 60.6% (95% CI: 55.9–65.2) when assessing photographs of men. The two-point percentage difference (62.5–60.6) was not statistically significant (p = 0.25).

We tested whether the participant’s response to the different questions depended on the sex of the persons in the photograph. The interaction effects between the traits and the sex of the persons in the photographs were not statistically significant [column H1b, rows ii2–ii4], and we also failed to reject the joint hypothesis of all interaction coefficients being zero (Wald test, F_4,79_ = 0.54, P = 0.71). Taken together, these results suggest that Tsimane’ adults were equally likely to attribute greater strength to taller adult women or to taller adult men in the photographs. However, they were not statistically more likely to attribute more dominance or knowledge to the taller adult in the photograph than to the shorter one.

Row IIA1 (column H1a) of Table 1 suggests that women were 7-percentage points more likely than men to select the taller person in the photographs when asked about any of the traits (p = 0.003). To estimate interaction effects between the participant’s sex and the traits being evaluated we ran four additional regressions to re-estimate the parameters in column H1a, but added an interaction term trait*female, with female being the participant’s sex. These additional regressions are not shown, but we found no significant interaction effects.

Hypothesis-2: *Shorter women will be perceived as more caring than taller women, particularly by male raters.* Results in column H2a (row IAi3) of Table 1, suggest that when asked about social concern, participants were not significantly more likely to select the shorter person over the taller one than when asked whether the taller or the shorter person was more knowledgeable (p = 0.79). This result does not hinge on our choice of the trait used as a reference group. We re-estimated the regression in column H2a but used dominance instead of knowledge as the base group; participants were not more likely to choose the shorter person over the taller one as having more social concern (p = 0.59; regression results not shown). We use the results in column H2b, row IIB1, to assess whether male raters were more likely to associate the shorter woman in the photograph with greater social concern than female raters. We found that female raters were 8-percentage points more likely than male raters to attribute greater social concern to the taller women, but results were no statistically significant (female*social concern; p = 0.15). However, we reject the null joint hypothesis that the coefficients for female (β = 0.07, p = 0.05) and the interaction term female*social concern (β = 0.08, p = 0.15) are zero at the 1% level. The Wald test for the joint hypothesis produced an F statistic (F_2,79_) of 6.00, with a p<0.01. Women raters were ∼15-percentage points more likely than male raters to attribute greater social concern to taller women in the photographs (column H2b, row II, Table 1).

To illustrate what this means, in analysis not shown we computed the predicted probability for female raters and found that the probability that female raters attributed more social concern to taller women in photographs was 63% (95% CI: 56–69), whereas the probability that male raters attributed more social concern to women in the photographs was only 47% (95% CI: 41–54). We re-estimated the two regressions for H2 (columns H2a and H2b in Table 1) using only photographs of men to assess whether shorter men were viewed as having more social concern than taller men, and whether there was a difference between female and male raters in their perception of social concern. We found no statistically significant results (β_female_ = 0.03; p = 0.22; β_female*concern_ = 0.05; p = 0.27; Wald test, F_2,79_ = 1.88; p = 0.16). In sum, we found some evidence that female raters were significantly more likely to choose the taller woman in the photograph as having more social concern than male raters.

Hypothesis-3: *Adults will evaluate taller children as stronger, more competent, and more knowledgeable than shorter children of the same sex and age.* Column H3a (section IAi) of Table 1, suggests that adult raters (both women and men) were more likely to associate taller girls and boys with greater strength, dominance, and knowledge [rows IAi1, 2, 4], than when rating tall versus short children for social concern. Raters were 11-percentage points (p = 0.001), 5-percentage points (p = 0.02), and 8-percentage points (p = 0.005) more likely to select the taller child as being stronger, more dominant, and more knowledgeable (p = 0.005), compared to the probability of choosing the taller child as more socially concerned.

To illustrate the magnitude for each trait independently, we estimated the predicted probabilities of choosing the taller child for an average Tsimane’ rater. When asking about strength, the predicted probability of choosing the taller girl was 65.6% (95%CI: 60.1–70.0), and the predicted probability of choosing the taller boy was 64.4% (95%CI: 59.6–69.2). The predicted probabilities of choosing the taller girl and boy for dominance were 59.4% and 58.3% (95%CI girl: 55.3–63.5; boy: 54.1–62.4), and for knowledge the predicted probabilities of choosing the taller girl and boy were 61.8% and 60.6% (95%CI girl: 57.3–66.2; boy: 56.9–65.4). However, for none of the four traits were these differences between girls and boys statistically significant at the 5% level.

We examined whether the sex of the participant rating the photographs of children [column H3a, row IIA1, Table 1] bore a significant association with their answers by interacting the sex of the rater with each of the four traits. These results are not shown. We found no significant results (female*____: strength, p = 0.13; dominance, p = 0.56; knowledge, p = 0.14; joint effects: Wald test, F_4,79_ = 1.36; p = 0.25). Interaction effects between the four traits and the sex of the child in the photograph were not statistically significant, indicating that the positive associations with height did not differ for boys or for girls (boys*_____: strength, p = 0.93; dominance, p = 0.56; knowledge, p = 0.21.; joint null hypotheses: Wald test, F_4,79_ = 0.49; p = 0.75) [Section IAii, Table 1]. In sum, results suggest that adult raters were significantly more likely to attribute more strength, dominance, and knowledge to taller girls and boys in the photographs, with no significant difference by the child’s sex.

We did additional analyses to ensure robustness (not shown). First, to reduce multicollinearity we eliminated all control variables: raters’ age, position of the taller person in the photograph, duration of test, and village of residence. We found essentially the same results. The only difference we found was that the variable for the participant’s sex was no longer statistically significant at the 5% level in one case (row IIA, column H2b: new coefficient = 0.05, SE = 0.03, p = 0.10). Second, we eliminated induced responses (1% of 7,627 responses), and third, we included controls for the surveyors, date and time of day when we administered the test to partially remove the role of the venue where the experiment took place. None of these changes affected the results.

## Discussion

Like their peers in industrial countries, adult Tsimane’ attributed some positive traits to the tall, but this was most marked when judging children, not adults. Tsimane’ adults attributed greater strength, dominance, and knowledge to taller girls and boys; the sex of the rater or the sex of the child did not affect results.

Unlike their peers in industrial countries, Tsimane’ did not attribute most positive traits to tall adults, but were significantly more likely to judge taller adults as stronger compared to other traits. The attribution of strength to the tall may partly explain the higher wages that tall workers receive in regions that rely mostly on manual labor [29], [30], or even preferences in mate choice [55]. In previous studies among the Tsimane’ we found weak associations between adult height and indicators of well-being [33], and low but positive assortative mating for height [56]. The attribution of greater strength to the taller person applied both to adult men and women, consistent with findings in industrial nations [1]. Whereas previous research in industrial nations [1], [22] suggests that both women and men perceive taller women as more masculine and less expressive or caring than shorter women, Tsimane’ women were more likely to attribute greater social concern to taller women, with no effect for Tsimane’ men. Overall, the absence of a strong association between adult height and desirable traits suggests that perceptions of height might mirror the actual benefits of height in a society.

Our results raise a puzzle: Why would Tsimane’ adults attribute most positive traits to tall children, but not tall adults? We can suggest three potential explanations for the finding. One possibility is that Tsimane’ are changing their perceptions about the benefits of height as they move from a self-sufficient economy and highly endogamous society to a market economy where people more commonly interact with strangers. As Sear and Marlowe note [57], in a small-scale, inward-looking, closely-knit society, people might not need to rely on height as a marker of unobserved traits because they can use more reliable markers (e.g., first-hand experience or experience with someone’s close relatives). As societies grow in size, heterogeneity, and complexity, people might need to rely more on markers of unobserved traits, with height being one such marker. As the Tsimane’ society opens up to more trade and other forms of interaction with the rest of the world, adults might associate height with desirable outcomes–particularly if outsiders are taller and are perceived as successful, or if logging and cattle-ranching operations prefer to hire taller individuals–but the positive attribution to the tall applies to the young who will face a new society. This explanation is consistent, for example, with Lee Cronk’s observations among the the Mukogodo of Kenya [37]. He found strategic increased investment in female compared to male offspring based on a changed social-economic context in which females were more successful on the marriage market than males. Closely related, some research has suggested that being short might be more adaptive in tropical rainforests, since it would provide advantages in hunting and gathering [58]. As Tsimane’ gain a stronger foothold in the market, being short may no longer provide such advantages.

A second possibility is that because low-income rural societies, such as the Tsimane’, are more commonly affected by contagious diseases, high parasite loads, and unpredictable food supplies [59]–[61], linear growth is an important indicator of good health and potential for survival among children. Child growth stunting is widespread among native Amazonian populations, including the Tsimane’ [43], [44]. Growth in height–particularly in the developing world–is strongly correlated with overall well-being [62]. Adults likely ascribe positive attributes to height in children because it is in their experience an indication of good health, particularly in this high-pathogen environment. A shorter child may signal lower capacity for work or weaker cognitive skills [63]–[65]. In contrast, by the time people reach adulthood, variation in height is going to be less closely tied to day-to-day variation in health, and–as Sear and Marlowe [57] note–individuals are able to base their assessments of desirable traits on direct observations.

Last, and related to the previous explanations, it is also possible that children show more variation than adults in the traits we assessed. Since Tsimane’ have very poor estimates of exact age, in part because many lack birth certificates [45], [47], adults may attribute traits to children based on their prediction of the child’s developmental stage using height as a more reliable indicator than age.

The study has several limitations. First, the height differential between the people in the photographs might not have reached the threshold to influence the judgments of raters. Tsimane’ adults display small variation in height, perhaps because they are a small-scale society of only ∼8,000 people that follows relatively strict endogamic rules through preferential cross-cousin marriage which may promote dense kinship networks that even out the distribution of resources [42], [66] and more similar genetic propensities. Correcting for age shrinkage, the average adult woman is 151.0 cm (SD: 4.8cm) tall, and the average adult man is about 162.9 cm (SD: 4.8cm) tall. Elsewhere we show that adult Tsimane’ have not experienced a significant secular change in standing physical stature during the 20th century [45]. Combined, all of this evidence suggests that variation in height among Tsimane’ is not large and as a result we could not vary the height of the people in the photographs too much, otherwise they would have appeared unnatural. We modified the photographs trying to emulate the variation of height one observes among the Tsimane’, but we did not use any specific algorithm or scale to generate the differences in height. Second, the question about social concern was problematic. We asked about the propensity of caring for a juvenile monkey. Participants might have interpreted the question as being about a concern for animal well-being more than a concern for other human beings. In our field observations, Tsimane’ often show little concern with the physical pain of non-human animals. Since the Tsimane’ regularly hunt wild animals (including monkeys) and for subsistence eat animals they live in close proximity with, they may develop different sensibilities about animal welfare than those in industrialized contexts who mainly encounter animals as pets and companions. Maybe some participants viewed shorter women as having more social concern, but they did not attribute to them greater concern for wildlife. Third, our manipulation of photographs probably introduced some distortions in body proportion, particularly among children. By changing height we may have inadvertently changed body mass–the taller people looked broader as well. Last, we focused on socioeconomic traits rather than on health or reproductive success. The positive correlation between height and good health or reproductive success might be more universal and provide sharper results than other attributions.

On a policy note, adult Tsimane’ attributed positive traits to tall children, perhaps because of the prevalence of growth-stunting in this high-pathogen environment. Indeed, perhaps it is because Tsimane’ parents can observe the health consequences of linear growth stunting that they ascribe greater value to height among children than adults. If people did not attribute positive traits to tall children, then one could have argued that one impediment to child growth stunting was cultural–parents not assigning enough importance to growth faltering because they did not associate child height with desirable outcomes. But that is clearly not the case here. Our results instead lead back to structural and ecological causes of growth faltering, such as poor nutrition, sanitation, and access to health care.
